# Transcend socioeconomic status constraints to mathematics and science achievement by collaborative problem-solving: The female people-smartness hypothesis

**DOI:** 10.3389/fpsyg.2022.944329

**Published:** 2022-08-26

**Authors:** Mei-Shiu Chiu

**Affiliations:** College of Education, National Chengchi University, Taipei City, Taiwan

**Keywords:** collaborative problem-solving, female people-smartness hypothesis, mathematics, science and reading achievement, PISA, socioeconomic status

## Abstract

This study examines the *female people-smartness* (FPS) hypothesis, which addresses the reasons why females are more responsive to socioeconomic status (SES) and posits that using females’ strengths of people-smartness can assist females to overcome SES constraints. This study used data from the student surveys of the Program for International Student Assessment (PISA) in 2015, including 519,334 students from 72 participating countries and economies. The results of the general linear model analysis revealed that females are better at collaborative problem-solving (CPS) and reading, while males are better at mathematics and science. Structural equation modeling revealed that the effect of SES on (mathematics and science) achievement is higher for females than for males. CPS can reduce the effect of SES on achievement. The findings generally support the FPS hypothesis and suggest that CPS-related competences should be emphasized and exercised to transcend SES constraints, especially for females in STEM curricula, studies and careers.

## Introduction

The persistent under-representation of females in science, technology, engineering, and mathematics (STEM) has raised the issue of adaptive educational design to increase females’ choice to study STEM (European Commission, 2019); Organization for Economic Cooperation and Development (OECD, 2019). Traditionally perceived, the key to gender differences in STEM choice is gender differences in STEM achievements (Good et al., 2008). With the concern of parental and socioeconomic factors in partially determining children’s STEM achievements (Penner and Paret, 2008), gender differences in problem-solving processes with potential for educational intervention are elected as a key to reducing gender differences in STEM achievement and participation (Zhu, 2007).

One problem-solving process that may distinguish females from males is the *female people-smartness* (FPS; vs. *male things-smartness*) hypothesis. The FPS emphasizes females’ interpersonal intelligence as an essential, critical, and unique competence. FPS may address the issue of females’ relatively lower achievements in almost all human societies across cultures and especially in STEM, with STEM emphasizing “things” and forming one of the major parts of the conventional, scholastic intelligence (Peterlin et al., 2021). Narrow ways to define (general) intelligence, such as intelligence quotient (IQ), may limit intelligence to academic fields, which emphasize solving well-structured problems independently and quickly in traditional tests.

Recent development in intelligence research has broadened the scope to include qualitatively different, multiple intelligences, which leads to suggestions for personalized, adaptive teaching (Gardner, 1995; Shearer, 2018). Emphasis on interpersonal intelligence and complex problem-solving in daily life also leads to the development and implementation of a test on collaborative problem-solving (CPS) by the Program for International Student Assessment (PISA) study in 2015 (OECD, 2017). The content of the PISA CPS test appears to comprise several, diverse 21st-century competencies (e.g., collaboration and problem-solving; Messersmith, 2015) and multiple process-related intelligence (Sternberg and Karami, 2021).

The purpose of this study, therefore, is to examine the FPS hypothesis using CPS as an indicator of interpersonal intelligence or people smartness. The following literature review will further explain the FPS using empirical studies, followed by three likely ways to examine the FPS hypothesis (i.e., FPS as strength, weakness, and power) as suggested by related studies. Given females’ under-representation in STEM, special focuses will be placed on gender differences in mathematics and science achievements, SES’s effect on achievements, and CPF’s power to reduce SES’s effect on achievements. A comparison will also be made with conventional academic (e.g., reading, mathematics, and science) achievements, competencies, and variables in IQ tests if CPS can also be viewed as an achievement, competence, or likely human intelligence (smartness).

### The FPS hypothesis

The FPS hypothesis speculates that females are sensitive to and thus smart at complex issues involving people and themselves as insiders (e.g., social interaction and communication). The FPS may become a convenient vehicle for females to learn. Males, on the other hand, are sensitive to things or objects (including people as a “thing” or “human source”) in the environment. Males are smart to study and evaluate things based on an absolute law or males themselves (also a thing).

Females’ sensitivity to people versus males’ sensitivity to things can manifest in gender differences in reflections on their experiences. Females write more online messages about their interactions with other people, while males write more about personal emotions in response to societal events (Barrett and Lally, 1999). Females recall their social interaction or communication with others in the community or platform when solving gamified mathematical problems, while males recall obstacles, scores, and tricks, directly relating to the games or the results of the problem-solving (Ke, 2008). The gender differences occur in females’ reflections on interpersonal relationships or social interactions, and males’ reflections on objects in the environment and related personal responses to the objects.

For a larger world or domains of knowledge, females express consideration of, responding to, and interest in people, while males express interest in things, mathematics, science, and engineering (Hyde, 2014). Girls prefer to become health or social-science professionals, which directly involve people; boys prefer science, engineering, and information and communication technology (ICT) disciplines, which more relate to things (OECD, 2015, pp. 119, 125; Su and Rounds, 2015).

The mechanism underlying the FPS may be that females tend to perceive themselves as part of the people or have a sense of belonging (Allen et al., 2021). Females, therefore, evaluate themselves relatively among each other and immerse themselves in the learning community, which may be based on intimacy goals as being related to females’ adaptive help-seeking (Kiefer and Shim, 2016). Collaborative cognition activation, therefore, can raise females’ interest in learning mathematics (Cantley et al., 2017). Males, on the other hand, perceive people as objects, resources, or things, evaluating all people (including themselves) on an absolute scale (e.g., justice). As evidenced, with dominance goals, males have fewer adaptive help-seeking behaviors but more expedient help-seeking behaviors (Kiefer and Shim, 2016).

The FPS hypothesis extends to three further sub-hypotheses. FPS as strength highlights females outperform males in people-related tasks. FPS as weakness indicates females’ sensitivity to social norms, hints, or environment. FPS as power states the function of FPS, if well-developed and -exercised, can overcome weakness and pursue achievement.

### Female people-smartness as strength: Gender differences in achievements

Gender differences in achievement across diverse academic domains have long been a research topic for approaching educational equality (Wang and Degol, 2017). Ideally, boys and girls should have equal abilities or achievements in different domains of knowledge, known as the gender similarities hypothesis (Else-Quest et al., 2010). Empirical studies, however, fail to fully support this. Some research finds that boys have higher achievements in mathematics and science, while girls have slightly higher achievements in languages (Robinson and Lubienski, 2011), although there are diversities across cultures (Chen and Zimmerman, 2007).

The 21st-century competencies have gradually emphasized competencies beyond traditional academic subjects (e.g., mathematics, science, and languages) and extended to non-academic competencies such as communication, cooperation, and solving complex (e.g., financial and daily-life) problems (Messersmith, 2015). Multitasking (an example of solving complex problems) appears to be a general phenomenon for both digital natives and immigrants (Evans and Robertson, 2020). Females appear to be equipped with multitasking tendencies, given females’ concerns about interacting with people while solving cognitive problems (Ke, 2008). This, however, may be a problem (e.g., execution, orientation, and attention; Wu and Cheng, 2019) or an opportunity if we see this from a different perspective such as a fully developed and functioning CPS competence.

This phenomenon can be extended to solving complex daily-life problems. For example, to resolve moral dilemma problems, males’ moral development more follows the route toward justice, against social power by self or ideal good, while females’ moral development is driven by interpersonal good, toward gratitude, responsibility, self-scarification, or caring (Gilligan, 1977). Males’ valuing justice is a virtue of “things,” which follow an absolute law, ruler, or self to achieve the absolute good. Females’ caring is a virtue for “people,” which emphasizes active interactions between human beings, including females themselves, in the community to achieve social good. The differential processes in resolving moral-dilemma issues manifest qualitative differences between genders in solving complex cognitive problems or social issues.

We can further infer that with complex problems involving both things and people, females’ people-smartness if fully functioning would likely facilitate their solving traditionally things-focused problems. The inference is based on the rationale that incorporating personal interests into teaching would increase student interest and achievement (Høgheim and Reber, 2015; Bernacki and Walkington, 2018; Clinton and Walkington, 2019). If females emphasize and value people (as the FPS hypothesis implies females’ interest in people), incorporating people-related pedagogies (e.g., CPS, collaborative/cooperative learning, and microteaching) into mathematics and science curricula might invite females to STEM fields.

### Female people-smartness as weakness: Relationships between socioeconomic status and achievement

Research generally finds positive, medium relationships between SES and academic achievements (e.g., STEM and reading) across diverse cultures (Hong and You, 2012; Tucker-Drob and Harden, 2012; Yoshino, 2012). Females’ PISA CPS scores are also more sensitive to economic inequality in societies than males at the country level (Wu et al., 2022); that is, economic inequality negatively predicts CPS for both genders, with females having a higher absolute slope in the predictive relationship than males. Socioeconomic factors, both at the individual level like family educational sources and at the group level like country wealth, are likely to be part of the reasons for the positive relationships between SES and achievement.

A reasonable guess based on the FPS hypothesis proposed by this study is that females are smarter, more sensitive, and more actively respond to social matters (e.g., SES) in their contexts. As such, the relationships between SES and achievements may be stronger for females than for males. Partial evidence for supporting the claim of FPS as a weakness is that females are more likely to experience a sense of being outsiders in STEM studies and careers than males (Master and Meltzoff, 2020). This self-representation of little belonging may be due to females’ gender stereotype threats in STEM, which in turn affect their STEM interest and achievement. Females’ sense of belonging mediates their social interaction perception leading to an interest in science, while males’ social interaction perception can directly lead to interest without the mediating effect of a sense of belonging (Allen et al., 2021). This self-representation of little sense of belonging may distract females from STEM interests and achievements.

### Female people-smartness as power: Transcend SES constraints

When there is a strength if fully developed and applied, the strength becomes a power, which may overcome constraints. As addressed, FPS may be a strength for females but also a weakness due to females’ sensitivity to social matters (e.g., SES). If females can fully develop and apply their FPS, females’ well-developed and -applied FPS would break the relationships between SES and achievement, especially in STEM.

A scientific methodology to provide evidence for FPS’ power to break SES-achievement relationships may be to examine the mediating effect of FPS. If the statistical path effects from SES leading to achievement can be reduced by FPS, then FPS can be seen as having the power to overcome SES constraints in students’ achievement. This methodology has been widely used by related studies. For example, SES can mediate the path relationship from technology-use patterns leading to achievement in mathematics, reading, and science (Chiu, 2020). With the FPS hypothesis, we can predict that there are gender differences in the degree of FPS’ mediating effects on the relationship from SES leading to achievement. Or, more precisely, the mediating effect of CPS on the capacity of SES predicting achievement is likely to be stronger for females than for males.

If we see CPS as a new competency, it is worth comparing CPS with other competencies, especially those represented by the achievements of the academic subjects in school. The high relationships in achievement scores between the major academic subjects in school (e.g., mathematics, science, and reading) lend support to the conception of general intelligence or IQ (Shearer, 2018; Peterlin et al., 2021). Domain specificity, on the other hand, justifies multiple intelligences (Gardner, 1995) and dimensional comparison theory (Möller and Marsh, 2013) for explaining individual differences in talents, career choices, and perceptions to form a collaborative human community. Cross-disciplinary learning can rely on this commonality and specificity between different domains of knowledge, skills, or competencies. In other words, we can assume competency transferability from one domain if two non-identical domains share some commonalities. Using statistical terms, transferability is mediating. Different competencies may play mediating roles in each other given their commonalities and specificities. CPS is a new competency highlighted in the 21st century in addition to the traditional academic competencies (mathematics, science, and reading). This study appears to be the first to address the mediating role of CPS, as a competency of transferability, and thus needs to make a comparison with the mediating roles of the traditional three academic subjects in school.

### Hypotheses

This study aims to examine the FPS hypothesis. Collaborative problem-solving (CPS) achievement serves as a proxy competence indicator of *people-smartness or interpersonal intelligence* in this study. CPS is part of the PISA 2015 test, inviting students to solve academic tasks with two virtual teammates by using three competencies: building team understanding, taking team actions, and maintaining team progress (OECD, 2017). Studies examining CPS’s validity reveal that the PISA CPS scores have moderate relationships with the scores of (student self-report, teacher-report, and co-player) collaborations and reasoning (Stadler et al., 2020); the personality traits of openness to experiences and agreeableness can predict the PISA CPS scores (Stadler et al., 2019). CPS as part of the essential 21-century competences may be understood more fully if compared with conventional academic achievements, competencies or variables in IQ tests (e.g., reading, mathematics, and science). Specifically, this study examines three hypotheses.

**Figure 1:**
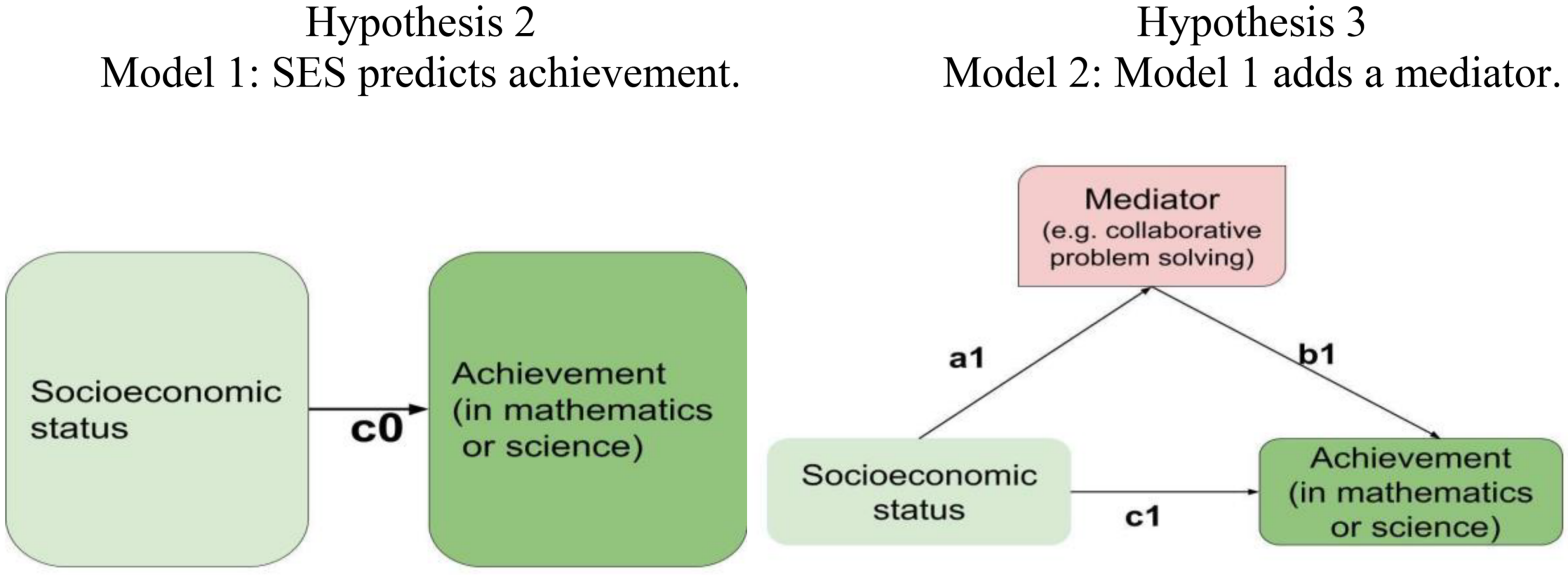
The proposed models for Hypotheses 2–3. The notations of the path coefficients (*c*0, *a*1, *b*1, and *c*1) follow the conventional notations used by Baron and Kenny (1986).

1. Females are better at CPS than males.
2. Socioeconomic status (SES) predicts mathematics/science achievement (Model 1 in Figure 1), especially for females.
3. The predictive capacity of SES on mathematics/science achievement is mediated or reduced by CPS (Model 2 in Figure 1), especially for females.

For Hypotheses 2–3, two models are posited and examined (Figure 1). SEM Model 1 posits a path relationship that SES predicts achievement in mathematics or science. SEM Model 2 adds a mediator to Model 1. Two methods are used to examine a mediating effect. First, the multiplication of the two path coefficients relating to the mediator (i.e., a1*b1, where a1 = the coefficient from SES to the mediator and b1 = the coefficient from the mediator to achievement) is significant. Second, the path coefficients directly from SES to achievement decrease from Model 1 to Model 2 (i.e., *c*0–*c*1 > 0).

## Materials and methods

### Data source and sample

This study used student data from the PISA 2015 study (OECD, 2016). PISA is a triennial study starting from 2000, collecting diverse achievement and related contextual data from 15-year-old students in grade 7 or above. PISA takes turns placing emphasis on reading, mathematics, and science. The PISA 2015 was the sixth round of study, mainly focusing on science and partially on mathematics, reading, financial literacy, and collaborative problem-solving (CPS).

In total, 72 participating countries and economies participated in PISA 2015. The total sample size was 519,334 students (50.1% females; 49.9% males).

### Measures

SES was a composite score of home possessions and parental highest vocation and educational levels. Students’ CPS, mathematics, reading, and science achievements were estimated using students’ responses to cognitive tests, scaled using item response theory (mean = 500; standard deviation = 100).

### Data analysis

For Hypothesis 1, a general linear model examined gender differences in the four achievements. The survey package for R set the survey design, and the mitool package dealt with plausible values of the four achievements.

For Hypotheses 2–3, the R intsvy package obtained population correlations among SES and the four achievements, which served as an initial understanding of bivariate relationships between the measures. Structural equation modeling (SEM) without or with mediating effects was performed using R survey, lavaan, and lavaan.survey packages, which took account of the sample survey design. All the correlation and SEM analyses were performed for all, female, and male students, separately.

This study focused on examining mediating roles. To simply the statistical procedure, the method to examine the mediating effect was path analysis using the SEM methodology, rather than the traditional full SEM with both path (regressions) and measurement (factor loadings) models. The SEM path model (without constraints) leads to a saturated path model, with a zero degree of freedom; that is, the number of parameters estimated in the model equals the number of the means, variances, and covariances of the observed variables (Raykov et al., 2013). A saturated path model, therefore, fits the covariance/means matrix of the raw data only. The traditional overall fit indexes all the root mean square error of approximation (RMSEA) = 0.000 and the comparative fit index (CFI) or [Tucker–Lewis index (TLI) = 1.000] cannot evaluate a saturated model but local fit (e.g., case residuals presented as plots) may be an alternative. The regression coefficients obtained by saturated SEM-based path models remain trustworthy and robust, as has been successfully used by related studies (Raykov et al., 2017; Chiu et al., 2021). This study chose to use significant regression (mediating) coefficients as a sign of local fit. The codes of all the analyses were shared on Kaggle.^1^

## Results

### Gender differences (Hypothesis 1)

All the gender differences in the four achievements are significant. Females have higher CPS [mean (*M*) = 498.152]; [standard error (SE) = 0.286] than males (*M* = 473.025; SE = 0.407; Table 1). The result supports Hypothesis 1 that females are better at CPS.

**Table 1:** Gender differences in four achievements.

	Female		Male		Female–male	
	Mean	SE	Mean	SE	Mean difference	*Z*
Mathematics	459.580	0.281	466.862	0.407	7.283	17.879
CPS	498.152	0.286	473.025	0.407	−25.127	−61.786
Reading	479.028	0.244	452.263	0.322	−26.765	−83.077
Science	468.659	0.221	470.360	0.309	1.701	5.510

All the *Z*s are significant at *p* < 0.05. SE = standard error. CPS = collaborative problem-solving.

Females also have higher reading achievement (*M* = 479.028; SE = 0.244) than males (*M* = 452.263; SE = 0.322). Males have higher mathematics achievement (*M* = 466.862; SE = 0.407) and science achievement (*M* = 470.360; SE = 0.309) than females (mathematics: *M* = 459.580; SE = 0.281; science: *M* = 468.659; SE = 0.221). There are larger gender differences for CPS (mean difference (MD) = −25.127; *Z* = −61.786) and reading (MD = −26.765; *Z* = −83.077) than for mathematics (MD = 7.283; *Z* = 17.879) and science (MD = 1.701; *Z* = 5.510). These results partially support Hypothesis 1 because of a large gender difference in CPS.

### Correlation (preliminary analysis before SEM)

Correlation analysis provides bivariate relationships between the measures. Correlations can provide an insight into the results of gender difference tests and a basic understanding of the relationships among the variables in SEM models (e.g., Models 1–2 in Figure 1). The analysis reveals that SES has moderate correlations with the four achievement measures (Table 2). The correlations between SES and the four achievements are stronger for females (*r*s = 0.435 to 0.450) than males (*r*s = 0.373 to 0.459).

**Table 2:** Correlations between the measures for all, female and male students.

	SES	Mathematics	CPS	Reading
*All students*				
Mathematics	0.410			
CPS	0.399	0.734		
Reading	0.417	0.812	0.783	
Science	0.426	0.893	0.811	0.888
*Female students*				
Mathematics	0.435			
CPS	0.435	0.745		
Reading	0.450	0.819	0.778	
Science	0.454	0.892	0.817	0.892
*Male students*				
Mathematics	0.384			
CPS	0.373	0.745		
Reading	0.394	0.827	0.784	
Science	0.399	0.894	0.822	0.900

Compared with the other three achievements, CPS tends to have the lowest correlations with SES. The correlations among the four achievements are large (*r*s = above 0.734; Table 2). CPS has the lowest correlations with the other three achievements (0.734 to 0.822), compared with the correlations between mathematics, reading, and science (0.812 to 0.900).

### Socioeconomic status predicts achievement (Hypothesis 2)

The SEM Model 1 (Figure 1) posits that SES predicts achievement. For the all student sample, the predictive relationships from SES leading to achievement are supported for both mathematics (path coefficient, *c*0 = 0.435) and science (*c*0 = 0.430; Table 3).

**Table 3:** Path coefficients based on Figure 1’s SEM analysis.

		Model 0		Model 1					Female-	Male
SES predicts	Mediator	*c*0	*a*1	*b*1	*c*1	*a*1*b1	Total1	*c*0–*c*1	*a*1*b1	*c*0–*c*1
**All**										
Maths		0.435								
	CPS		0.399	0.679	0.319	**0.271**	0.413	**0.116**		
	Reading		0.437	0.775	0.096	0.339	0.435	0.339		
	Science		0.43	0.886	0.063	*0.372*	0.435	*0.372*		
Science		0.430								
	CPS		0.399	0.762	0.122	**0.304**	0.426	**0.308**		
	Reading		0.437	0.859	0.054	0.376	0.430	0.376		
	Maths		0.435	0.871	0.051	*0.379*	0.430	*0.379*		
**Females**										
Maths		0.448								
	CPS		0.435	0.686	0.137	**0.298**	0.435	**0.311**		
	Reading		0.464	0.782	0.085	0.363	0.448	0.363		
	Science		0.448	0.864	0.061	*0.387*	0.448	*0.387*		
Science		0.448								
	CPS		0.435	0.764	0.122	**0.332**	0.454	**0.326**		
	Reading		0.464	0.863	0.048	0.376	0.430	*0.400*		
	Maths		0.448	0.863	0.061	*0.487*	0.448	0.387		
**Males**										
Maths		0.421								
	CPS		0.373	0.700	0.123	**0.261**	0.384	**0.298**	*0.037*	**0.013**
	Reading		0.420	0.795	0.088	0.334	0.421	0.333	**0.029**	*0.030*
	Maths		0.412	0.870	0.063	*0.358*	0.421	*0.358*	**0.029**	0.029
Science		0.412								
	CPS		0.373	0.782	0.108	**0.291**	0.399	**0.304**	0.041	**0.022**
	Reading		0.420	0.876	0.044	0.368	0.412	0.368	**0.008**	*0.032*
	Maths		0.421	0.878	0.042	*0.370*	0.412	*0.370*	*0.117*	0.017

All the path coefficients (*c*0, *a*1, *b*1, and *c*1), mediating effects (*a*1*b1), and total effects (total1) are significant at *p* < 0.05. For mediating effects “*a*1*b1” and “*c*0–*c*1,” italic numbers indicate the highest values and bold the smallest among the three mediators.

For both females and males, SES can solely predict mathematics/science achievement (*c*0s = 0.412 to 0.448 in Table 3; Model 1; Figure 1). In terms of the path coefficient values, the capability of SES predicting achievement is stronger for females (mathematics: 0.448; science: 0.488) than for males (0.421; 0.412). The results support Hypothesis 2: family SES predicts achievement, and the predictive capacity is stronger for females than for males.

A note to make is that multigroup SEM-based path models examined Model 1 (Figure 1) gender differences failed to provide trustworthy results for answering Hypothesis 2. It was because multigroup SEM for examining Model 1 resulted in non-identified models (non-positive definite variance–covariance matrix of the estimated parameters). Both configural invariance (all parameter estimates being set equal for female and male groups) and weak invariance (only regression parameter equal) obtained the same results. As such, this paper does not report multigroup SEM results (The code and results are present on Kaggle).^2^

### Mediating effects (Hypothesis 3)

#### All students

The SEM Model 2 adds a mediator to Model 1 (Figure 1). All the path coefficients (*c*0, *a*1, *b*1, *c*1), mediating effects (*a*1*b1), and total effects (total1) are significant (Table 3). In addition, the results (obtained by two methods) support that there are mediating effects for all the mediators. The first method uses direct mediating effects; that is, a multiplication of the two path coefficients relating to the mediator (from SES and to achievement, i.e., *a*1*b1) are all significant. The second method is that the path coefficients from SES to achievement reduces from Model 1 to Model 2 (i.e., *c*0–*c*1).

Comparisons are made between the mediators (i.e., CPS, reading, mathematics, and science) in their mediating effects obtained by both methods (i.e., *a*1*b1 and *c*0–*c*1). CPS’s mediating effects are the weakest (*a*1*b1 = 0.271 for mathematics and 0.304 for science; *c*0–*c*1 = 0.116 and 0.308). Reading is in the middle (0.339, 0.376; 0.339, 0.376). Science achievement (0.372; 0.372) and mathematics achievement (0.379; 0.379) are the strongest to mediate the effects of SES on mathematics and science achievement, respectively. This may be due to the similarity in mathematics and science competencies, as indicated by the higher correlations between maths and science (Table 2).

#### Female and male students

For both girls and boys, the effect of SES on mathematics/science achievement can be mediated by CPS, reading, and science/mathematics achievement (*a*1*b1 = 0.261 to 0.487; *c*0–*c*1 = 0.298 to 0.400; Table 3; Model 2 in Figure 1). For gender differences, the mediating effects are larger for females. As shown in the last two columns of Table 3, all the values for “Female–Male” on “*a*1*b1” and “*c*0–*c*1” are positive.

In the direct mediating effects (*a*1*b1) on SES predicting mathematics and science achievements, CPS has the largest gender difference (mathematics: 0.037; science: 0.041; Table 3). The next is science/mathematics (0.029; 0.117), followed by reading (0.029; 0.008).

In the reduced path coefficient coefficients (*c*0–*c*1), the largest gender difference occurs in reading (0.030 for mathematics; 0.032 for science). The next is science/mathematics (0.029; 0.017), followed by CPS (0.013; 0.022).

The results reveal that all mediating effects are stronger for females than for males, which appears to imply that females need more or diverse competencies to overcome the constraints of SES. This finding appears to be new in literature. Although this guess is not part of the hypotheses, this issue deserves future research.

## Discussion

### Female people-smartness as strength: CPS favors females

The result of the general linear model analysis support Hypothesis 1 that females are better at collaborative problem-solving (CPS) than males. The results support the female people-smartness (FPS) hypothesis, proposed by this study, based on past related studies (Ke, 2008; Hyde, 2014; OECD, 2015). CPS is the strength of females, compared with males.

The FPS hypothesis partially suggests a male things-smartness (MTS) hypothesis. As such, this study extends gender difference tests on CPS achievement to conventional academic (reading, mathematics, and science) achievements, which are more ‘things’ than “people.” The test results find that girls are better at reading and worse at mathematics and science than boys. If we use the MTS to explain the results, males’ higher achievements in mathematics and science imply that mathematics and science are more “things,” while reading is slightly far away from “things.” The results are consistent with past findings that males’ higher achievement in mathematics and science, though not supported universally (Guiso et al., 2008; Else-Quest et al., 2010). Mathematics and science as “things” further explain why males have higher mathematics and science interest and career expectation, compared with females’ higher interest and career expectations in social and health sciences (Hyde, 2014; OECD, 2015). This result partially supports the posited FPS hypothesis and the inferred MTS hypothesis.

Correlations between the four achievements may provide insights into reading as being more a thing or interpersonal competence and how CPS relates to the other three competencies. Reading has higher relationships with mathematics (all: 0.812; female: 0.819; male: 0.827; Table 2) and science (0.888; 0.892; 0.900) than with CPS (0.783; 0.778; 0.784). CPS has the lowest correlations with mathematics (0.734–0.745), followed by reading (0.778–0.784) and science (0.811–0.822).

The above results of correlations suggest that CPS appears to be a unique competence that may be distinguished from mathematics, science, and reading. Mathematics, science, and reading have long been three major academic competencies in national curricula worldwide and one of the major variables in IQ tests (Shearer, 2018; Peterlin et al., 2021). CPS appears to be a competence undervalued in national curricula and intelligence research. CPS is also unique and a likely operational representation of interpersonal intelligence, a meaningful outcome by emotional intelligence (Mayer et al., 2008; MacCann et al., 2020), or a combination of multiple intelligences (Gardner, 1995), which need to be researched and considered to be emphasized in national curricula.

### Female people-smartness as weakness: SES impacts achievement, especially for females

Positive relationships between SES and achievement are a persistent phenomenon worldwide (Tucker-Drob and Harden, 2012; Yoshino, 2012). This study’s finding supports the phenomenon that for the overall student sample of this study, SES predicts both mathematics and science achievements. Further, the effect of SES on achievement is more substantial for females than males.

This result appears to support the FPS hypothesis functioning in a negative direction. In the current human society, there still exists gender inequality disfavoring females in society across diverse cultures (Heise et al., 2019; Dahal et al., 2021), also in STEM (Sattari and Sandefur, 2019). Females’ sensitivity to social cues, conformity to social structures, and hesitation to break social norms may further worsen their weak status in society.

The above claim is supported by research results that females experience greater gender stereotype threats, and feel like outsiders in STEM (Master and Meltzoff, 2020). A piece of partial evidence is that gender gaps in STEM achievement diminish in gender-equal societies (Guiso et al., 2008; Hyde, 2014), although different tests with different samples may show different results (Else-Quest et al., 2010; Reilly et al., 2019). The findings of this study encourage the development of social measures to diminish SES constraints for females entering STEM careers, as part of indicators for gender-equal societies.

### Female people-smartness as power: CPS transcends SES constraints, especially for females

One unique finding of this study perhaps is that for all students, CPS can reduce SES constraints on mathematics and science achievements. The reason for this may be that CPS (interpersonal intelligence or people smartness) is competency and has relatively high but non-identical relationships with mathematics and science (Table 2). People-smart individuals may use their interpersonal intelligence to overcome the constraint of SES for better science and mathematics achievement. Better help-seeking behaviors and interactions with peers and teachers in the learning environment are found positively related to achievement (Schenke et al., 2015). CPS, therefore, is a competency worth pursuing and being included in the spectrum of school curricula.

For gender differences, all the CPS’s mediating effects are much more substantial for females than for males. As shown in Table 3’s last two columns, all the values for “Females–Males” in the two kinds of mediating effects are positive. Compared with gender differences in the mediating effects of the conventional academic competencies (in reading, mathematics, and science), gender differences in CPS’s mediating effects are the strongest for mathematics and moderate for science; the results, however, are based on significant direct mediating effects (*a*1*b1) only, the not reduced path coefficients (*c*0–*c*1 > 0). The FPS hypothesis posited in this study can explain the gender differences in CPS’s mediation effects. If one of the females’ strengths is CPS (Hypothesis 1), then a fully functioning CPS can be the moving power for females to overcome SES constraints (Hypothesis 2) and pursue achievements (e.g., in mathematics and science, as posited by Hypothesis 3). The result is consistent with research findings that adolescent females seek more help from peers and teachers than their male counterparts (Schenke et al., 2015). The mechanism for females’ CPS strength may root in both biological and social factors that drive females to focus more on people and skills in interpersonal interactions or communications.

Though not the focus of this study, there are the largest gender differences in the mediating effects of mathematics and reading. The reasons may be rooted in the commonality of the academic domains (e.g., IQ; Shearer, 2018). Cross-domain mediating effects are with educational interest in the era emphasizing multi- or cross-disciplinary learning and deserve future research. The different results obtained by the two kinds of mediating effect methodologies (direct coefficients and reduced path coefficients) in this study suggest future research to clarify and advance methodologies for mediating effects.

The result supporting CPS’ mediating effects appears to be new in the literature and offers the potential to improve educational practices by CPS curricula and pedagogies for inviting females’ participation in STEM study and careers. Further, CPS appears to include multiple 21st-century competencies (Messersmith, 2015), though with a special focus on collaboration or interpersonal intelligence. Perhaps multiple disciplines (or cross-domain) learning or educational design involving multiple intelligences (Shearer, 2018) would be more suitable for females.

For educational practices, it may be worth reconsidering whether national curricula should separate school hours according to disciplinary subjects. Traditional teaching and society emphasizing individual achievement, independence, and competitiveness may also underestimate or undermine females’ achievements and development, especially for STEM careers, and should be amended. The advance in ICT has made lifelong learning for multiple disciplines accessible, feasible, and possible, which may offer opportunities to develop diverse competencies. Future ICT-infused pedagogical design may need to focus on developing diverse competencies simultaneously (e.g., CPS), especially for STEM subjects and females.

### Contributions, limitations, and suggestions for future research

#### contribution

This study proposes the female people-smartness (FPS) hypothesis, which highlights females’ interpersonal intelligence. The FPS hypothesis is evidenced by females’ higher CPS achievements and higher CPS mediating effects to transcend females’ stronger link of socioeconomic status (SES) to achievement. The stronger link between SES and achievement can be viewed as females’ weakness due to females’ being more responsive or vulnerable to societal constraints (e.g., SES). Precisely, this study supports the FPS hypothesis by providing evidence for FPS as a strength, weakness, and power for females. The findings suggest that more educational provision should be placed on increasing students’ CPS competencies, especially for females’ participation in science, technology, engineering, and mathematics (STEM) education or careers.

#### Limitations and suggestions

This study used data from an international database, which may increase the generalization of the findings, but cultural differences may still be an issue to be resolved by future research. CPS as an indicator of people-smartness or interpersonal intelligence is reasonable in terms of its meaning and operation in the PISA’ CPS test. Other indicators may be created in daily life settings (e.g., management and leadership). Future research needs to consider the effect of reading on females. It is because reading is also a strength for females and serves as a good mediator to reduce the link of SES leading to achievement.

SES is a relatively long-standing nurturing environment pre-determined by parents or closely related others for students’ development. The long-existing essence of SES allows for examining the predictable capacity of SES for achievement (Hypothesis 2) by path analysis in statistical terms, especially given PISA only collects cross-sectional data. Mediating effects can serve as an alternative for the assuming cause-effect (or actual path) relationship, as used in this study (Hypothesis 3). However, only a randomized experimental-control design can properly examine a cause-effect relationship. A longitudinal design is a next choice for examining SES’s effect, given that SES is hard to be an independent variable in experiments.

## Data availability statement

The datasets presented in this study can be found in online repositories. The names of the repository/repositories and accession number(s) can be found at: PISA 2015 study (OECD, 2016).

## Author contributions

The author confirms being the sole contributor of this work and has approved it for publication.

## Conflict of interest

The author declares that the research was conducted in the absence of any commercial or financial relationships that could be construed as a potential conflict of interest.

## Publisher’s note

All claims expressed in this article are solely those of the authors and do not necessarily represent those of their affiliated organizations, or those of the publisher, the editors and the reviewers. Any product that may be evaluated in this article, or claim that may be made by its manufacturer, is not guaranteed or endorsed by the publisher.

## Footnotes

^1^https://www.kaggle.com/code/meishiuchiu1/pisa2015genderclps-glm; ~-correlations; ~-semall; ~-semfemale; ~-semmale

^2^ https://www.kaggle.com/code/meishiuchiu1/pisa2015genderclps-semall#multigroup-SEM:-Model-0-between-genders
